# Circulating Biomarkers in Failing Fontan Circulation: Current Evidence and Future Directions

**DOI:** 10.3390/jcdd12090358

**Published:** 2025-09-16

**Authors:** Cecilia Vecoli, Lamia Ait-Alì, Simona Storti, Ilenia Foffa

**Affiliations:** 1 CNR Institute of Clinical Physiology, 54100 Massa, Italy; lamia.ait-ali@cnr.it (L.A.-A.); ilenia.foffa@cnr.it (I.F.); 2 Fondazione Toscana Gabriele Monasterio, 54100 Massa, Italy; storti@ftgm.it

**Keywords:** Fontan circulation, circulating biomarkers, artificial intelligence, machine learning, -omics approaches

## Abstract

Patients with Fontan circulation are at lifelong risk for a range of complications involving multiple organ systems. As survival into adulthood increases, there is an urgent need to refine strategies for long-term follow-up and the early detection of Fontan-related sequelae. This narrative review aims to provide a comprehensive summary of the current evidence regarding the use of circulating blood biomarkers as non-invasive tools for assessing and monitoring Fontan physiology. We critically analyzed available studies investigating serum biomarkers related to key pathological mechanisms associated with Fontan failure, encompassing not only cardiac dysfunction but also systemic inflammation, endothelial dysfunction, hepatic and renal impairment, and altered bone metabolism. Several biomarkers have shown promise in reflecting global systemic impairments as well as end-organ involvement in Fontan patients. However, current data are insufficient to support evidence-based clinical recommendations for standardized specific biomarkers, mainly due to the small sample sizes, heterogeneous patient populations, and limited longitudinal data in the available studies. Only a large-scale, prospective, multi-center, and multidisciplinary research will permit us to identify a panel of specific biomarkers of clinical utility in this population. Artificial intelligence (AI) and machine learning (ML) approaches could be applied to integrate all these heterogeneous datasets. Furthermore, “omics”-based studies, including proteomics, metabolomics, lipidomics, and microRNA profiling, hold great potential for uncovering novel biomarkers and pathophysiological pathways, ultimately paving the way for precision medicine in the management of Fontan patients.

## 1. Introduction

The functionally single ventricle represents the most complex congenital heart defect (CHD), affecting roughly 1 in 10,000 individuals [1]. The prevalence among adults continues to grow due to the success of the staged Fontan procedure. The first or second stage of palliation is usually the bidirectional Glenn procedure, in which the ligated superior vena cava is anastomosed to the confluent pulmonary arteries [1]. The Fontan circulation is then completed by redirecting blood from the inferior vena cava into the pulmonary arteries. This was initially performed with the atriopulmonary connection (APC), later modified into the lateral tunnel (LT), and ultimately refined as the extracardiac total cavopulmonary connection (TCPC) [2]. In the Fontan physiology, systemic venous blood flows passively into the lungs without the aid of a subpulmonary ventricle. In contrast, the single functional ventricle sustains the systemic output [3]. The circulation thus functions as a neo-portal system, where systemic and pulmonary capillary beds are arranged in series without an intervening ventricular pump [4]. The Fontan circulation represents a palliative surgical strategy with the main purpose of separating the systemic and pulmonary circulations in the absence of a subpulmonary ventricle. In this “novel” circulatory setting, deoxygenated blood moves passively to the lungs, while the single functional ventricle is solely responsible for maintaining the systemic output [1,2,5].

The Fontan operation, first described in 1971 for repairing tricuspid atresia [4], is now a palliative surgery that enables an increasing number of patients to survive into adulthood [6,7]. However, up to 50% of Fontan patients experience a major adverse event before reaching adulthood [3].

**Figure 1:**
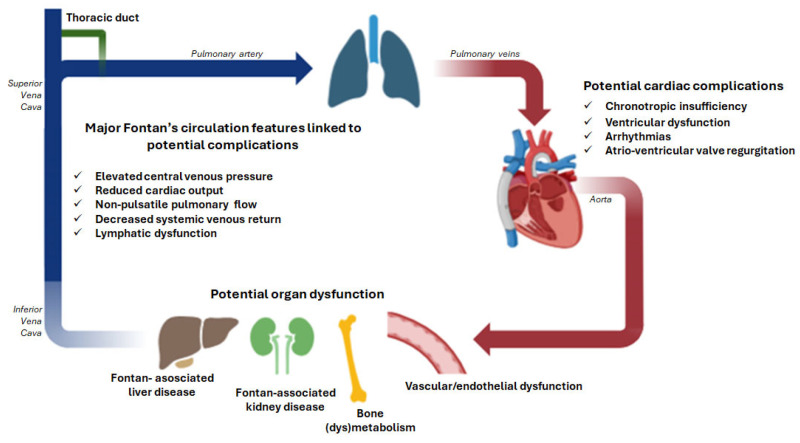
Schematic representation of pathophysiological mechanisms due to the Fontan circulation anatomy implicated in the multiorgan dysfunction reported in this review. This figure was partially created using BioRender (https://www.biorender.com/ on 2 September 2025).

The term Fontan “failure”, referring to the physiological dysfunction resulting from chronically elevated central venous pressure (CVP) and reduced cardiac output (CO) [8], is used to describe a broad spectrum of clinical manifestations that may occur in numerous ways with unpredictable timing [9]. The circulatory failure leads to multiorgan damage (Figure 1), primarily driven by chronic systemic venous hypertension resulting from the absence of a subpulmonary ventricle. However, the underlying pathophysiological mechanisms remain incompletely understood.

Therefore, there is a dual need: on one hand, to identify biomarkers capable of detecting early signs of Fontan circulatory failure; on the other, to determine biomarkers associated with the onset and progression of multiorgan dysfunction. This approach is essential to optimize follow-up strategies and improve the management of long-term complications in this unique population [7].

In 2019, the AHA Scientific Statement highlighted a critical gap in characterizing Fontan pathophysiology and proposed a cardiovascular surveillance testing scheme to drive the clinical decision-making [3]. Briefly, a range of diagnostic tools has been recommended for the follow-up of Fontan patients, including electrocardiography (ECG), transthoracic echocardiography, 24 h Holter monitoring, exercise stress testing, serum BNP or NT-proBNP levels, but also more advanced imaging and invasive investigations, including cardiac magnetic resonance imaging (MRI), computed tomography, and cardiac catheterization [3].

The 2020 ESC guidelines on adult congenital heart disease stated that catheterization should be considered early in the presence of clinical deterioration, such as reduced exercise capacity, unexplained edema, new-onset arrhythmia, cyanosis, or hemoptysis [1]. Nevertheless, hemodynamic parameters measured invasively (i.e., central pulmonary artery pressure or systemic ventricular end-diastolic pressure) show limited correlation with adverse outcomes [3], limiting the availability of dynamic parameters to accurately characterize the Fontan circulation. Consequently, there is a strong need for novel tools to evaluate Fontan circulation under everyday physiological conditions, along with validated markers that better reflect patient status and risk. In this context, the identification of circulating biomarkers that reflect the functional status of various organs and systems is of growing interest, as blood molecules have high clinical practicality, given that sampling is minimally invasive and commonly performed in clinical settings. Hence, circulating biomarkers offer a valuable opportunity for non-invasive monitoring, prognostic stratification, and potentially therapeutic guidance in the Fontan population. Unfortunately, the complex pathophysiology of these patients—characterized by reduced pulmonary pulsatile flow, chronically elevated central venous pressure, relative hypoxemia, and systemic inflammatory activation—complicates the interpretation of conventional laboratory markers.

In recent years, several studies have evaluated several biomarkers not only strictly associated with cardiovascular impairment but also involved in the pathophysiological mechanisms leading to global body failure in different cohorts of patients with Fontan circulation, seeking correlations with adverse clinical outcomes [5]. Biomarker-guided approaches would align with the principles of precision medicine and the development of individualized management pathways that reflect the heterogeneity of Fontan physiology.

**Figure 2:**
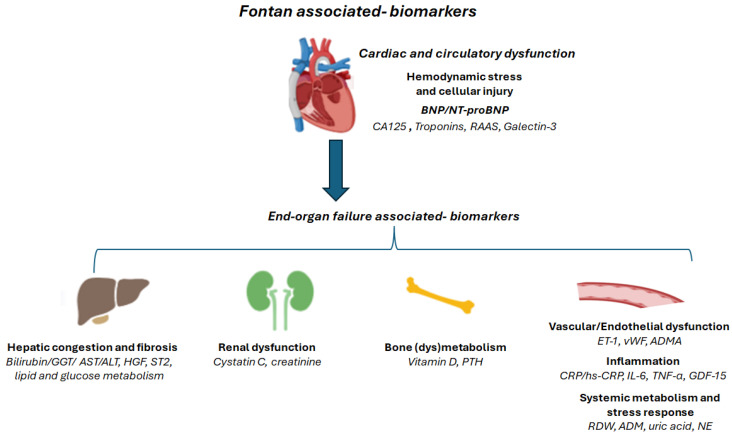
Schematic representation of circulating biomarkers associated with major potential complications related to Fontan failure, including cardiac and circulatory dysfunction (hemodynamic stress and cellular injury-associated biomarkers), hepatic congestion and fibrosis, renal dysfunction, bone (dys)metabolism, vascular/endothelial dysfunction, inflammation, as well as biomarkers linked to systemic metabolism and stress response. Cardiac dysfunction is strictly associated with abnormal levels of BNP/NT-proBNP–the only biomarker highly recommended in the management of Fontan patients by current guidelines [3]. This figure was partially created using BioRender (https://www.biorender.com/ on 2 September 2025). BNP: brain natriuretic peptide; NT-proBNP: N-terminal prohormone of brain natriuretic peptide; CA125: Carbohydrate antigen 125; RAAS: renin–angiotensin–aldosterone system; RDW: red blood cell distribution width; ADM: adrenomedullin; ET-1: endothelin 1; NE: norepinephrine; vWF: von Willebrand Factor; ADMA: Asymmetric dimethylarginine; CRP: C-reactive protein; hs-CRP: high sensitive-C-reactive protein, IL-6: interleukin-6; TNF-α: tumor necrosis factor; GDF-15: Growth/differentiation factor-15; HGF: Hepatocyte growth factor; ST2: suppression of tumorigenicity 2; GGT: gamma-glutamyl transferase; AST: aspartate aminotransferase; ALT: alanine aminotransferase; PTH: parathyroid hormone.

This review aims to provide an up-to-date overview of the principal circulating biomarkers investigated in post-Fontan patients, with a particular focus on their clinical significance and prognostic value (Figure 2). We also discuss potential future directions towards a personalized medicine framework.

## 2. Pathophysiological Pathways Reflected by Circulating Biomarkers

A biomarker is generally defined as a “characteristic that is objectively measured and evaluated as an indicator of normal biological processes, pathogenic processes, or pharmacologic responses to a therapeutic intervention” [10]. Biomarkers are generally classified into four categories: (1) diagnostic—for early disease detection; (2) prognostic—for predicting disease course; (3) predictive—for estimating treatment response; and (4) therapeutic—for identifying novel treatment targets [11]. Moreover, they may also serve as surrogate endpoints in clinical trials. The characteristics of biomarkers vary based on their intended use. Nevertheless, an ideal biomarker should meet several criteria: (1) it must be accurate, reproducible, easy to obtain, and inexpensive; (2) it must provide added value over existing measures; (3) it must aid in clinical decision-making [12].

In this section, we summarize the principal studies performed in cohorts of post-Fontan patients, presenting the available evidence in alignment with the key pathophysiological pathways that are currently recognized as being involved in, or altered during, the development and progression of Fontan failure. A more detailed discussion can be found in previous reviews by Rajpal et al. (2017) [13] and Wittczak et al. (2025) [5].

### 2.1. Hemodynamic Stress and Cellular Injury

Fontan failure encompasses a diverse range of clinical presentations, including heart failure (HF) and/or portal hypertension, which often necessitate Fontan revision or heart transplantation [5]. Like HF due to acquired cardiovascular disease in adult subjects, Fontan failure is progressive. However, while acquired HF is commonly secondary to hypertension, atherosclerosis, or other cardiovascular comorbidities, Fontan failure develops from the unique, chronic hemodynamic burden imposed by a passive, non-pulsatile pulmonary blood flow [5]. Furthermore, individuals with Fontan physiology often present supraventricular arrhythmias, a reduced aerobic capacity, cardiac fibrosis, and both diastolic and systolic dysfunction. Identifying circulating markers able to drive or early identify these sequelae is essential in the prevention of clinical adverse outcomes.

#### 2.1.1. BNP and NT-proBNP

Brain natriuretic peptide (BNP), a polypeptide hormone synthesized, stored, and secreted by cardiac muscle cells, plays a major role in many pathophysiological processes. Its blood levels have been shown to be a diagnostic and prognostic biomarker of cardiac dysfunction and/or heart failure [14]. Actually, in clinical practice, the concentration of the N-terminal prohormone of brain natriuretic peptide (NT-proBNP) is commonly measured because of its longer half-life [15].

The scientific statement of the American Heart Association on the “Evaluation and Management of the Child and Adult with Fontan Circulation” highly recommend the evaluation of serum BNP or NT-proBNP levels once in childhood, every 1–3 years in adolescents, and every 1–2 years in adults [3]; nevertheless, it issued no recommendation about the interpretation of results [3]. Thus, although natriuretic peptides are among the most widely studied biomarkers in adults with congenital heart disease, their usefulness appears to be limited in patients with Fontan circulation, as highlighted by the ESC Guidelines on adult congenital heart disease [1,16].

In 2004, Ohuchi et al. first identified the serum concentration of BNP as an independent factor able to classify patients according to NYHA class II and III/IV [16]. Ten years later, serum BNP concentration also resulted in an independent predictor of all-cause mortality in both pediatric and adult Fontan patients [17,18,19]. Other studies showed the value of BNP in monitoring the cardiovascular status and function [20,21]. Koch et al. (2008) found normal BNP levels in most Fontan patients in their population study up to 15 years after TCPC [22]. Still, elevated levels of BNP were linked to greater morbidity and late mortality [22]. Likewise, in a larger cohort (*n* = 510), Fontan patients exhibited generally BNP levels within the normal range [23]; however, their more elevated levels were linked to some indicators of poorer outcomes, although these correlations were relatively weak. Thus, the authors concluded that BNP might not be suitable for routine outpatient surveillance in asymptomatic patients [23]. More recently, in the study by Nguyen et al. (2020), BNP levels did not accurately reflect decompensated HF in Fontan patients when compared to non-Fontan HF patients, highlighting the limited reliability of BNP in decompensated Fontan failure [24]. Of note, these different results can be, at least in part, ascribed to the different techniques of surgery used for the Fontan procedure that may affect the release of BNP as well as NT-proBNP in the bloodstream.

NT-proBNP resulted in drastically high levels in pediatric Fontan patients with congestive heart failure (CHF), and a strong correlation with the severity of disease was observed. Fontan patients without CHF had NT-proBNP levels similar to healthy children [25], and the plasmatic level of NT-proBNP seems also related to the type of Fontan circulation: older types (i.e., atrio-pulmonary connection or atrio-ventricular connection) that involve more atrial tissue in the systemic venous pathway have shown higher NT-proBNP levels independently of their cardiac status compared to patients with TCPC. In patients with TCPC (but not patients with atrio-pulmonary connection or atrio-ventricular connection), the plasmatic concentrations of NT-proBNP positively correlated with atrioventricular valve and ventricular dysfunction [25].

NT-proBNP was found to be a fundamental marker for the identification of patients with significant ventricular dilation or dysfunction [26]. Moreover, the inverse association between serum NT-proBNP levels and dyssynchrony measurements, and exercise capacity suggested that these parameters should be investigated further in Fontan patients [27]. The levels of NT-proBNP might have major utility also as a prognostic marker during the follow-up period [28,29,30]. In 2021, van den Bosch et al. [31] found that NT-proBNP levels were significantly associated with adverse outcomes, also after adjustment for age, sex, and ventricular dominance, identifying it as the most useful biomarker for risk stratification [28]. Perrone et al. (2022), in a study performed to evaluate the beneficial effects of a 4 4-week aerobic exercise program on cardio-pulmonary performance in patients with HLHS and Fontan circulation, demonstrated that NT-proBNP levels significantly decreased together with the soluble form of suppression of tumorigenicity 2 (sST2) after 4 weeks of aerobic exercise program [32]. This change in biomarker profile was associated with an improvement of cardio-pulmonary performance in adult Fontan patients with hypoplastic left heart syndrome (HLHS) [32].

Recently, Buendía Fuentes et al. (2023) investigated the role of NT-proBNP and several other biomarkers with respect to clinical complications in a prospective study involving 66 Fontan patients [33]. In this study, thrombocytopenia, high NT-proBNP, RDW (red blood cell distribution width), and CA125 (carbohydrate antigen 125) levels, as well as liver fibrosis indices FIB4 (fibrosis 4) and APRI (AST to Platelet Ratio Index), were associated with a worse clinical profile. However, only RDW, CA125, and FIB4 remained associated with Fontan circulation failure in the multivariate model [33]. Although further studies are needed to confirm this conclusion, the authors highlighted the specific role of CA125, a known marker of systemic congestion and inflammation in patients with HF [34], which, for the first time, has been identified as a useful biomarker in the evaluation of Fontan circulation failure, a condition in which venous congestion plays a central role.

#### 2.1.2. Troponins

Troponins are essential regulatory proteins involved in the contractile apparatus of striated and cardiac muscle that are released into the bloodstream in response to myocardial injury. Hence, they serve as sensitive and specific biomarkers of cardiac damage [35], but they also have prognostic value in heart failure [36]. In the myocardium, the troponin complex consists of three subunits: troponin C (TnC), troponin T (TnT), and troponin I (TnI). In the Fontan population, the levels of troponins are inconsistent between various studies [5]. Nevertheless, a recent systematic review stated that patients with Fontan circulation (in contrast to other patients with a different CHD) had low hs-TnT (high sensitive troponin T) levels, probably because of their particular circulation that led to a relatively underloaded ventricle, which might result in a smaller myocardial injury [37].

#### 2.1.3. The Renin-Angiotensin-Aldosterone System (RAAS)

A number of studies investigated the role of the renin–angiotensin–aldosterone system (RAAS) that represents a key endocrine/paracrine network modulating some cardiovascular functions [38]. In the initial stages of heart failure, RAAS activation serves as a compensatory response; however, as the disease advances, this activation becomes maladaptive, contributing to elevated preload and afterload [38]. High renin activity was associated with diuretic use and low arterial pressure, suggesting a specific role of RAAS in maintaining perfusion pressure in stable Fontan patients [16]. Moreover, RAAS activation resulted in a trigger of hypovolemia [39]. Inai et al. (2005), in a population of 50 Fontan patients, found a negative association of angiotensin II levels with left ventricular ejection fraction [40]. The authors ascribed angiotensin II as a marker of ventricular damage from volume overload and/or prior cyanosis [40]. Positive associations between RAAS components and procollagen type III N-terminal amino peptide (PIIIP) levels—a marker of fibrosis—were also reported in Fontan patients. Overall, this study supported the potential of serum PIIIP as a diagnostic marker for myocardial fibrosis, while also indicating that RAAS inhibition could contribute to the prevention of ventricular fibrosis after Fontan surgery [41].

#### 2.1.4. Galectin-3

Emerging evidence indicates that Gal-3 may serve as a biomarker of cardiac disease, including myocardial dysfunction and acute HF [42]. Galectin-3 (Gal-3), a roughly 30 kDa chimera-type galectin, is prominently expressed on the cell surface and actively secreted into biological fluids by injured and inflammatory cells. Adult Fontan patients showed higher plasma Gal-3 levels compared to matched control subjects [43]. Moreover, patients with high Gal-3 levels had an increased risk of adverse outcomes, including a higher risk of nonelective hospitalization or death [43]. In these Fontan patients, Gal-3 was positively correlated with age, uric acid, and hs-CRP and negatively correlated with estimated glomerular filtration rate (eGFR) [43]. Conversely, van den Bosch et al. (2021) found no association between circulating levels of this Gal-3 and clinical events or cardiac function parameters during follow-up [31]. Hence, its utility as a potential biomarker deserves further investigation.

### 2.2. Inflammation

Inflammation is the body’s natural defense mechanism against injury or infection and plays a central role in the pathogenesis of cardiovascular diseases, including HF [44]. Key processes involved in the inflammatory response include recognition of the initial insult, release of pro-inflammatory cytokines, and recruitment of immune cells to the site of injury. Recent studies have highlighted the undervalued role of inflammation and immune dysregulation in the context of CHD [45]. In particular, the practice of thymectomy during surgical correction of CHD has raised concerns due to its potential long-term effects on immune function and cytokine signaling [46].

#### 2.2.1. C-Reactive Protein

C-reactive protein (CRP) is a pentameric protein synthesized by the liver in response to inflammation. In the clinical setting, CRP functions as an acute-phase reactant, primarily induced by IL-6 acting on the hepatic gene responsible for CRP transcription during inflammatory or infectious processes [47]. The hs-CRP assay is a quantitative analysis test of very low levels of CRP in the blood. Generally, post-Fontan patients with higher values of hs-CRP have shown a higher risk of adverse outcomes [48,49].

#### 2.2.2. Pro-Inflammatory Cytokines (IL-6, TNF-α)

After the Fontan procedure, the levels of pro-inflammatory cytokines, including IL-6 and TNF-α, have been found elevated [49,50,51] with a parallel decrease in anti-inflammatory cytokine IL-10 [51], suggesting that Fontan patients might be characterized by a chronic subclinical inflammation [52]. High levels of inflammation were also found as a potential candidate mechanism of protein-losing enteropathy (PLE), a severe and potentially life-threatening complication following Fontan operation characterized by enteric protein loss [53]. Moreover, Miyamoto et al. (2016) evaluating a panel of circulating biomarkers in a population of 103 adult patients with CHD (including 53 with Fontan circulation), found that high levels of IL-6, hsCRP, and sTNF-RI 3 (the extracellular domains of the TNF receptor) significantly correlated with mortality in a univariate analysis in patients with a left ventricular morphology of systemic ventricle [18].

Saraf et al. (2020), in addition to elevated levels of IL-6 and TNF-α, found that GDF-15 (growth/differentiation factor-15), a member of the transforming growth factor-β (TGF-β) cytokine superfamily, was significantly increased in Fontan patients with clinical events [52]. Moreover, GDF-15 correlated with longer duration of Fontan and was elevated in atrio-pulmonary Fontan circulation [52]. Elevated serum levels of GDF-15 were also associated with worse functional status and predictive of adverse outcomes [54]. More recently, Perrone et al. (2022) evaluated the beneficial effects of a 4-week aerobic exercise program on cardio-pulmonary performance in patients with HLHS and Fontan circulation [32]. They reported that the most interesting data was the significant increase in GDF-15 [32]. Notably, GDF-15 is not solely a marker of cardiac stress but also reflects systemic responses to various forms of physiological stress and metabolic regulation. The authors observed that the increase in GDF-15 after the 4 weeks of training might reflect a physiological adaptation to the training program, indicative of beneficial muscle stress and improved metabolic regulation [32].

### 2.3. Endothelial Dysfunction

Endothelial dysfunction appears to be a consistent feature across all stages of single ventricle CHD palliation, although the precise timing of its onset remains unclear. Abnormal endothelial function has been documented in Fontan patients and has been associated with impaired cardiac output during orthostatic stress testing [55]. Endothelial dysfunction may influence multiple aspects of clinical progression of Fontan patients—including exercise intolerance, hepatic congestion, thromboembolic events, pregnancy outcomes, transplant candidacy, and ultimately Fontan failure—but the causal relationships have yet to be established. Moreover, endothelial function has been proposed as a promising therapeutic target [56] even if its longitudinal variation and prognostic significance in the Fontan population remain poorly defined.

#### 2.3.1. Endothelin-1

Endothelin-1 (ET-1), a peptide primarily produced by endothelial cells of blood vessels and cardiomyocytes, has prothrombotic and pro-inflammatory effects with a pivotal role in the development of various cardiovascular diseases. Evidence has highlighted high ET-1 concentration in the blood of Fontan patients [57,58,59,60]. In the study by Kolcz et al. (2011), a significant correlation of ET-1 and proBNP levels with VE/VCO2 peak was found to indicate these molecules as useful for the identification of high-risk Fontan patients [29]. Of note, although more studies are needed to confirm the utility of ET-1 as a biomarker, the ET-1 receptor antagonists (such as bosentan) are recommended by the ESC ACHD guidelines (class 2b, level C) in Fontan patients with increased pulmonary pressure/resistance [1].

#### 2.3.2. Von Willebrand Factor (vWF)

Von Willebrand factor (vWF) is a large multimeric glycoprotein produced mainly by endothelial cells. It has a major role in facilitating platelet adhesion and aggregation at sites of vascular injury, and in transporting coagulation factor VIII (FVIII) in the bloodstream [61]. vWF is stored in Weibel–Palade bodies that are released upon endothelial damage; for that, it is considered as a biomarker of endothelial dysfunction. More studies measured vWF in patients with Fontan circulation, showing its increase as well as its association with an elevated risk of thrombosis [62,63]. Notably, van den Bosch et al. (2021) found higher vWF levels associated with a worse severe event-free survival, although no thromboembolic events were registered [31].

#### 2.3.3. Asymmetric Dimethylarginine (ADMA)

Asymmetric dimethylarginine (ADMA), an endogenous inhibitor of endothelial nitric oxide synthase (eNOS), competes with L-arginine at the active site of eNOS and promotes endothelial cell apoptosis. Increased ADMA levels were found to be associated with endothelial dysfunction in both healthy individuals and those with cardiovascular disease [64], as well as with reduced exercise capacity in adults with CHD [65]. Fontan patients showed high levels of ADMA [66].

### 2.4. End-Organ Involvement: Hepatic Congestion and Fibrosis

The passive venous return directly into the pulmonary circulation increases arterial oxygen saturation, leading to a significant rise in CVP. All that results in several extracardiac consequences. including the Fontan-associated liver disease (FALD) that has emerged as a notable comorbidity of Fontan circulation [67]. Multiple contributing factors—including perioperative insults, chronic hypoxia, low cardiac output, and elevated CVP—may drive this unique form of congestive hepatopathy, which is characterized by sinusoidal and portal fibrosis and may progress to cirrhosis and portal hypertension [68,69,70,71].

Diagnosis of FALD is challenging due to the absence of reliable physical exam findings, relatively normal laboratory values, and the lack of pathognomonic imaging features, and liver biopsy remains the gold standard for diagnosis [71]. Surveillance biopsy series have consistently demonstrated that nearly 100% of young adult Fontan patients exhibit histologic evidence of liver fibrosis [68,69]. More recently, it has become evident that FALD can progress to cirrhosis or hepatocellular carcinoma, posing additional challenges in managing young adults who may also require heart transplantation due to Fontan failure [71]. Despite efforts to identify laboratory markers as well as imaging features, or catheterization variables associated with fibrosis severity, results have been limited.

#### 2.4.1. Bilirubin, GGT, AST and ALT

Regarding the more classical liver parameters such as bilirubin, gamma-glutamyl transferase (GGT), aspartate aminotransferase (AST), and alanine aminotransferase (ALT), evidence shows that FALD differs from other hepatic diseases, including viral hepatitis. In Fontan patients, the increase in AST and ALT is usually mild, while GGT or total bilirubin levels are often increased [72]. More studies reported increased levels of ALT, AST, GGT, and direct bilirubin levels in patients after the Fontan procedure, respectively [72]; in particular, high GGT levels were significantly associated with FALD after the Fontan procedure [73,74]. The elevation of GGT was likely caused by the epithelial injury of the bile ducts secondary to reduced vascular supply to the intrahepatic biliary system, reflecting hepatic congestion consequent to the Fontan procedure. High GGT levels were significantly associated with higher ALT concentrations and decreased platelet counts, findings suggestive of fibrosis progression. Moreover, treatment with ursodeoxycholic acid (UDCA) was associated with a reduction in GGT levels. These results underscore the clinical relevance of elevated GGT levels in patients with FALD [74].

#### 2.4.2. Hepatocyte Growth Factor

Hepatocyte growth factor (HGF), a mesenchymal cytokine with a pivotal role in the development of various epithelial and endothelial cells, is released after endothelial injury [75]. HGF resulted in an increase in Fontan patients with PLE compared to those without PLE, suggesting a major role of HGF in the onset of PLE after a Fontan operation [76]. Notably, higher values of HGF (at multivariate analysis) were able to independently predict both elevated CVP and decreased arterial oxygen saturation in a population of 34 Fontan patients [77]. Moreover, the receiver-operating characteristic (ROC) curve analysis indicated that HGF > 0.405 ng/mL (with 75% sensitivity and 83.3% specificity) resulted also a good index for additional catheter intervention after Fontan surgery [77].

#### 2.4.3. Suppression of Tumorigenicity 2

The suppression of tumorigenicity 2 (ST2) is a protein involved in a number of pathophysiological processes, especially related to inflammatory and autoimmune disease [78]. This protein exists in two different forms: one transmembrane (ST2 ligand) and one soluble (sST2). The sST2 amount is recognized as a helpful biomarker for risk stratification in chronic HF [79]. Since, compared to BNP, sST2 exhibits lower biological/analytical variability and its levels are not influenced by age or renal function. For these characteristics, it has been included in ACC/AHA guidelines, including sST2 dosage for improving risk stratification in patients with acute and chronic left HF [80].

In patients with Fontan circulation, sST2 levels were among the most critical predictors of acute heart failure [81]. Of note, in the already cited study by van den Bosch et al. (2021), higher levels of sST2 were associated with severe adverse events during follow-up [31]. Higher plasmatic levels of sST2 were confirmed by Geenen et al. (2019) in a cohort of 38 patients with Fontan circulation [82]. Accordingly, Perrone et al. (2022) registered a significant reduction in sST2 levels after a 4-week exercise program in 12 Fontan patients [32].

#### 2.4.4. Cholesterol Metabolism

Evidence reported that hypocholesterolemia (defined as lipoprotein levels below the fifth percentile of the general population adjusted for age, sex, and race) is commonly found in patients with Fontan circulation [83,84,85]. In particular, hypocholesterolemia indicates a more severe liver dysfunction, and high-density lipoprotein cholesterol (HDL-C) levels may be the most consistent indicator of the degree of liver disease [85]. More specifically, hypocholesterolemic state seems to be due to an increase in cholesterol absorption rather than decreased cholesterol synthesis [84]. In 2021, Lubert et al. [85] found that low HDL-C was associated with adverse events, likely driven by chronic inflammation and influenced by the same determinants observed in the general population, as male sex and increased body mass index [85]. Interestingly, in this Fontan cohort, HDL-C correlated with higher ALT and lower albumin but not with other markers of hepatic disease severity, including Model for End-stage Liver Disease excluding INR (MELD-XI) or VAST score (Varices, Ascites, Splenomegaly, and Thrombocytopenia, used to assess the severity of portal hypertension). More recently, Lu et al. (2024) reported that liver fibrosis was associated with decreased platelet counts and cholesterol levels, suggesting the appearance of hepatic functional deterioration [86]. In 2020, Michel et al. [87] performed a detailed analysis of small organic molecules in extended phospholipid and acylcarnitine metabolic pathways in adult Fontan patients. They showed that each class of lipid (phosphatidylcholine, lysophosphatidylcholine, sphingomyelin, and acylcarnitines) was decreased in Fontan patients compared to age- and sex-matched [87].

#### 2.4.5. Glucose Metabolism

The exact pathophysiology of abnormal glucose metabolism in Fontan patients is incompletely understood, and it is not excluded that it may have different causes, including myopenia, since skeletal muscle is a major consumer of plasma glucose. However, evidence suggests that hepatic dysfunction might largely contribute to the abnormalities in glucose homeostasis observed in Fontan patients [88]. Adult Fontan patients had lower fasting glucose, but HbA1c (a marker of long-term glycemic status) and C-peptide (a marker of endogenous insulin production) were both increased [89]. A large study showed that abnormal glucose metabolism progressed even in young adult Fontan patients [88]. In this cohort of patients, HbA1c had a poor predictive value for progression, whereas the oral glucose tolerance test played an important role in uncovering unique Fontan-associated abnormal glucose metabolism as well as predicting all-cause mortality [88].

### 2.5. End-Organ Involvement: Renal Dysfunction

As the Fontan population continues to grow, cardiorenal syndrome has emerged as a significant yet often underrecognized complication, reflecting the complex bidirectional interplay between cardiac and renal dysfunction [90]. The exact mechanisms contributing to renal impairment in patients with Fontan circulation remain incompletely understood. However, it is plausible that central venous pressure is elevated and chronically reduced cardiac output directly impacting renal function. Since early childhood, their kidneys operate under persistently high venous pressure and suboptimal perfusion, leading to progressive structural and functional impairment. By late adolescence or young adulthood, about 20% of patients develop mild renal impairment, and around 10% progress to moderate chronic kidney disease. While mild dysfunction may initially be tolerated, concern increases as patients reach their 30 s–40 s due to the risk of accelerated renal decline [90]. Although some evidence indicates that mild renal dysfunction in Fontan patients may remain stable for several years—providing a potential window for intervention—Fontan-associated kidney disease is often recognized only at advanced stages, frequently coinciding with the onset of Fontan circulatory failure [90]. This delayed detection underscores the need for improved strategies and emphasizes the importance of early and systematic screening.

#### Cystatin C and Creatinine

Renal dysfunction (defined as an eGFR < 90 mL/min/1.73 m^2^ based on creatinine) is observed in approximately 10–20% of Fontan patients [90]. However, the use of creatinine-based estimation methods in this population has been questioned due to the high prevalence of sarcopenia, which may lead to an overestimation of true renal function. Indeed, no conclusive association has been established between creatinine-based eGFR and long-term adverse clinical outcomes in Fontan patients. In contrast, cystatin C-based eGFR appears to be a more reliable biomarker in this setting, as it is independent of muscle mass. Lower cystatin C-derived eGFR values have been more consistently associated with adverse outcomes following the Fontan procedure. Nevertheless, the utility of cystatin C is not without limitations, as its levels may be influenced by thyroid dysfunction, inflammation, and glucocorticoid exposure, conditions not uncommon in this patient population [91]. Given these considerations, a combined approach using both creatinine and cystatin C may provide a more accurate assessment of renal function in Fontan patients.

Cystatin C is a 13-kilodalton protein, constantly produced by all nucleated cells, filtered at the glomerulus, and metabolized in the proximal tubule. Cystatin C-based eGFR resulted significantly lower in the Fontan group compared with healthy controls and was associated with adverse outcomes (while creatinine-based estimates are not) [92]. Opotowsky et al. (2019) in a large cohort of adults with CHD, including also 131 Fontan patients, found that only cystatin C-based eGFR strongly predicted clinical events compared to creatinine-based eGFR in adult CHD; in particular, the authors opened a discussion on the limits of creatinine-based methods in the Fontan circulation [93]. Katz et al. (2023) confirmed that the eGFR may be overestimated by creatinine in the Fontan population, favoring the use of cystatin C-based eGFR [94]. However, on the one hand, cystatin C might be a useful biomarker of renal dysfunction in Fontan patients compared to creatinine since it estimates eGFR independent of muscle mass; but, on the other hand, it may be affected by thyroid dysfunction and glucocorticoid activity. Hence, in this moment, the best choice could be to screen renal function with both cystatin C and creatinine.

### 2.6. End-Organ Involvement: Bone (dys)Metabolism

Patients with Fontan palliation may have decreased bone formation and/or increased bone resorption, resulting in bone loss and ultimately osteoporosis due to low cardiac output and chronic, albeit mild hypoxia [95].

#### 2.6.1. Vitamin D

Vitamin D is a fat-soluble vitamin that is naturally present in a few foods, added to others, and available as a dietary supplement. It is also produced endogenously when ultraviolet (UV) rays from sunlight strike the skin and trigger vitamin D synthesis from 7-dehydrocholesterol or are ingested from food. Then, it must be activated to 25-hydroxyvitamin D (25-OH-vitamin D) and finally to its active form, 1,25-dihydroxyvitamin D [1,25(OH)2D] [96]. Vitamin D has a well-known role in musculoskeletal health, but increasing evidence has shown a strict association between low levels of vitamin D and increased risk of cardiovascular diseases [97]. In Fontan patients, vitamin D deficiency (<20 ng/mL) was found [98,99,100], especially in those patients with lower muscle mass in the legs [98], Diab et al. (2019) observed an age-related decline in both bone mineral density (BMD) and vitamin D levels [101], although the vitamin D level decrease was not associated with bone mineral densities. Vitamin D sufficiency also demonstrated a strong positive association with exercise performance. Weinreb et al. (2020) identified a positive association between vitamin D sufficiency and peak VO_2_ in individuals with Fontan circulation, suggesting that maintaining adequate vitamin D levels-possibly through regular physical activity-might contribute to improved maximal exercise capacity [102]. Moreover, sufficient vitamin D status could offer protective effects against sarcopenia, a major concern in this population. These findings imply that vitamin D supplementation and/or the promotion of consistent physical activity from a young age may provide long-term benefits in preserving muscle mass and enhancing functional capacity in Fontan patients.

#### 2.6.2. Parathyroid Hormone

Calcium and phosphate homeostasis in the blood is primarily regulated by the parathyroid glands. The parathyroid glands secrete the parathyroid hormone (PTH), a polypeptide, in response to low blood calcium levels. PTH promotes the production of active vitamin D in the kidneys. Together with vitamin D, PTH regulates calcium and phosphate balance by acting on bones, kidneys, and the small intestine. When serum calcium levels decrease, PTH secretion increases; conversely, elevated calcium levels trigger a negative feedback mechanism that suppresses PTH release. The regulation of calcium by PTH is complex, and disruptions in this system can have important clinical consequences. Elevated levels of PTH were independently associated with a higher risk of HF in the general population [103]. In the Fontan population, like vitamin D, PTH has been studied in relation to the musculoskeletal system. In the first study, bone and muscle deficits were not associated with PTH levels [99]. Later, studying the incidence of chronic kidney disease in patients with Fontan circulation, Sharma et al. (2016) found that PTH levels were significantly higher in Fontan subjects compared to healthy controls, suggesting the augmented prevalence of hyperparathyroidism as a marker of renal dysfunction [104]. Higher levels of PTH were also found in patients with PLE [100], strongly associated with systemic inflammation [100]. Finally, D’Ambrosio et al. (2019) reported a significant inverse correlation between PTH and corrected calcium and 25-OH vitamin D; conversely, a positive association was found between PTH and NT-proBNP level and aldosterone [95]. The authors assumed that PTH being elevated and negatively correlated with vitamin D levels was highly suggestive of secondary hyperparathyroidism associated with subclinical vitamin D deficiency [95]. This state may be linked to changes in calcium metabolism caused by an altered renal perfusion or inadequate absorption in the gut. However, the authors also highlighted the utility of the treatment of vitamin D deficiency in Fontan patients to improve bone health and reduce the risk of fragility fractures.

### 2.7. Systemic Metabolism and Stress Response

Patients with Fontan circulation frequently exhibit alterations in systemic metabolism and stress-related pathways, reflecting the chronic hemodynamic burden and multisystem nature of the Fontan physiology. Alterations in systemic metabolism as well as the response to different stresses largely influence the general homeostasis, leading to a series of disturbances, including endothelial dysfunction and progressive end-organ alterations.

#### 2.7.1. Red Blood Cell Distribution Width

Anemia is particularly common in patients with complex CHD and ventricular dysfunction, especially those with Fontan, physiology which has been associated with increased mortality [105]. The etiology of anemia in adult patients following the Fontan procedure remains poorly understood, and its potential impact on functional capacity and exercise performance has yet to be adequately explored.

The red blood cell distribution width (RDW) is a measure of variation in the erythrocyte size (anisocytosis), automatically calculated in a standard complete blood count test (as the ratio between the standard deviation of the erythrocyte volume and the mean corpuscular volume). Higher values have been associated with higher mortality in heart failure [106]. In 2014, a study aimed at characterizing hematologic changes and iron metabolism observed that adult Fontan patients exhibited a higher RDW compared to control subjects [62]. The RDW also resulted in an independent predictor of oxygen uptake, suggesting a correlation of RDW with exercise tolerance at least in these patients. Thus, it is conceivable that RDW might serve as an index of iron deficiency and lower physical activity in adult patients with Fontan circulation [62]. Interestingly, in a retrospective study on 38 consecutive pediatric patients with a Fontan circulation, the RDW was a significant independent predictor of CVP and mixed venous oxygen saturation (SvO2). Contrariwise in the same population, the BNP levels showed no significant association with the CVP, SvO2, or cardiac index. The authors concluded that RDW was a convenient and powerful marker for detecting HF in Fontan patients [107].

#### 2.7.2. Adrenomedullin

The peptide hormone, adrenomedullin (ADM), has a pivotal role in vasodilatation as well as in the maintenance of endothelial function [108]. A first study published in 1999 reported lower ADM levels in the early postoperative setting after Fontan completion compared to control subjects [59]. In this study, the increased levels of ET-1 lead to an imbalance between ET-1 and ADM that could contribute to a dominant vasoconstrictor tone after the Fontan procedure [59]. Conversely, later studies in patients with Fontan circulation reported elevated levels of adrenomedullin (or its precursor peptide, the mid-regional pro-ADM) [109,110].

#### 2.7.3. Uric Acid

Serum uric acid (UA), the final metabolite of purine metabolism, has been closely associated with cardiovascular diseases. Uric acid causes inflammation and increases oxidative stress in vascular endothelial and smooth muscle cells, contributing to endothelial dysfunction [111]. Elevated UA levels may worsen coronary atherosclerosis, acute coronary syndromes, and hypertension, and are an independent predictor of mortality in both acute and chronic heart failure [112]. Hyperuricemia (defined as uric acid ≥ 7.0 mg/dL) was found in 22% of Fontan patients, with a higher prevalence in adult patients and reflected global postoperative Fontan pathophysiology, including the adverse prognosis (also with differences between children and adults); however, its prognostic value was not independent in the multivariate analyses [17].

#### 2.7.4. Norepinephrine

Norepinephrine (NE), one of the primary endogenous catecholamines released by the adrenal medulla as well as the central and sympathetic nervous systems, may act as a hormone and neurotransmitter. NE plays a critical role in modulating cardiovascular homeostasis [113]. In the context of HF, increased sympathetic nervous system activity leads to elevated circulating levels of NE, which in turn enhances myocardial contractility, induces peripheral vasoconstriction, increases heart rate, and elevates metabolic demand [114]. Impaired cardiac autonomic nervous activities and increased neurohumoral activities characterize Fontan patients. To date, there is almost no evidence of correlation between NE levels and Fontan clinical status. NE (in addition to BNP) appears to be useful in differentiating Fontan patients with NYHA II class from those with NYHA III+IV [16]. Elevated levels of NE were found in Fontan patients with low levels of sodium, a condition that predicts adverse clinical events [39]. Moreover, in a group of 91 Fontan patients, higher plasma concentration of NE was the only CANA (cardiac autonomic nervous activity) variable predictor of unscheduled cardiac events [115]. Finally, the combined assessment of plasma norepinephrine levels and peak oxygen uptake (VO_2_) provides prognostic insight in this population [40]. In children, NE, together with other parameters such as the use of diuretics, central venous pressure, liver enzymes, and plasma creatinine, was independently associated with uric acid, which, in turn, mirrored global postoperative Fontan pathophysiology [17].

## 3. Methodological Challenges of Biomarker Research in the Fontan Population

This review has outlined the available evidence on serum biomarkers in Fontan circulation, evaluating their association with specific pathophysiological sequelae. While numerous biomarkers have been proposed to characterize the pathophysiological complexity of the Fontan circulation, only a limited subset currently holds potential for clinical application. The interpretation of a role for a specific biomarker in the Fontan population is challenged by several methodological limitations. First, most available investigations are based on small, single-center cohorts, often with wide age ranges and heterogeneous surgical histories, which hinder reproducibility and generalizability. Second, study designs are predominantly cross-sectional, thereby limiting the ability to establish temporal relationships between fluctuations in biomarkers and clinical outcomes. Longitudinal studies that might allow the evaluation of temporal changes within the same population, offering a greater insight into disease course and supporting causal inference, remain scarce. Third, variability in laboratory methods and cutoff values further complicates the comparison of results across different cohorts. Altogether, these methodological variables may explain the multiple discrepancies between studies. In addition, clinical endpoints are inconsistently defined, ranging from surrogate measures of Fontan physiology to composite adverse outcomes, making it difficult to identify robust predictors. Finally, confounding factors such as medication use, comorbidities, and age-related changes in biomarker expression are often underreported or not adequately controlled. Addressing these methodological challenges will be crucial to advancing the field toward reliable, evidence-based biomarker applications in clinical practice.

## 4. Future Perspectives

### 4.1. From Single Biomarkers to an Integrated Biomarker Panel

**Figure 3:**
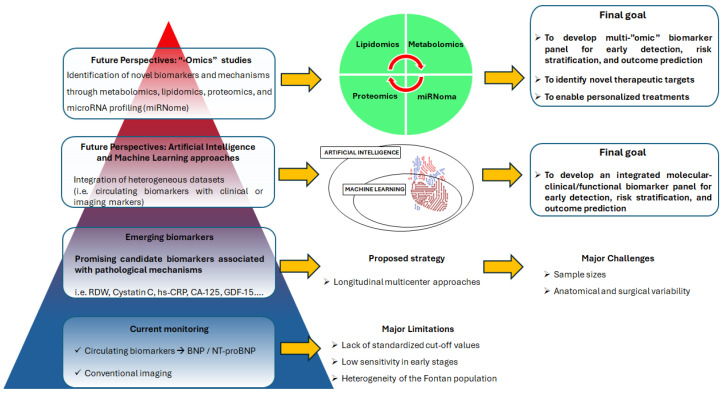
Schematic representation of the progressive evolution in biomarker-based monitoring strategies for patients with Fontan circulation. The pyramid structure illustrates three conceptual levels. Level 1(base): current clinical practice relies primarily on conventional biomarkers such as BNP and NT-proBNP, as well as standard imaging techniques, which lack sensitivity for early detection and have no standardized cut-off values specific to this population. Level 2: emerging candidate biomarkers, including RDW, cystatin C, hs-CRP, CA125, GDF-15, might enhance diagnostic and prognostic accuracy if investigated in longitudinal, multicenter studies. Level 3: Artificial intelligence and machine learning approaches will integrate heterogeneous datasets, i.e., circulating biomarkers and clinical/imaging data, with the goal of improving risk stratification and outcome prediction in complex conditions. Level 4 (apex): future perspectives involve multi-“omics” approaches such as proteomics, metabolomics, lipidomics, and microRNA profiling (miRNome), which offer promising opportunities to identify novel biomarkers, reveal previously unrecognized pathophysiological mechanisms, and support the development of precision medicine strategies tailored to the unique and complex Fontan population. BNP: brain natriuretic peptide; NT-proBNP: N-terminal prohormone of brain natriuretic peptide; RDW: red blood cell distribution width; hs-CRP: high sensitive C-reactive protein; CA125: carbohydrate antigen 125; GDF-15: Growth/differentiation factor-15.

The only biomarker recommended by current clinical guidelines is BNP/NT-proBNP [3], although a lack of robust, evidence-based cut-off values in this unique patient population remains. Other candidates, like sST2, already included in ACC/AHA guidelines for improving risk stratification in patients with HF [80], require further investigation in larger, longitudinal, and multicenter studies to confirm their clinical utility, sensitivity, and specificity in predicting Fontan-related complications. For instance, markers of hepatic or renal dysfunction may reflect advanced stages of end-organ impairment but often lack the sensitivity to detect early subclinical changes. This highlights the need for multiparametric approaches that integrate cardiac, hepatic, renal, endothelial, inflammatory, and systemic biomarkers into a unified risk-stratification model. From a translational perspective, biomarker research in the Fontan population offers the opportunity to bridge mechanistic insights with clinical decision-making. Biomarker panels that integrate end-organ and systemic pathways could serve as adjuncts to imaging and functional testing, improving risk stratification and refining follow-up strategies. Hence, a further promising strategy for the development of a comprehensive risk stratification model should incorporate both circulating biomarkers and clinical/functional parameters (i.e., diagnostic imaging markers). In this context, the growing complexity of datasets derived from all these parameters underscores the need for advanced analytic tools. Artificial intelligence (AI) and machine learning (ML) approaches are increasingly being applied to integrate these heterogeneous data collections, with the goal of improving risk stratification and outcome prediction in complex conditions [116]. By capturing nonlinear interactions between biomarkers and imaging features, AI-based models may uncover latent patterns that remain undetectable through conventional statistical methods. Such approaches could ultimately support precision medicine by enabling individualized follow-up strategies, facilitating the early identification of patients at high risk, and optimizing the timing of advanced interventions (Figure 3).

In 2022, a study by Truong et al. [117] investigated the association between several circulating biomarkers of myocardial fibrosis and myocardial fibrosis as detected by cardiac magnetic resonance using an ML approach. More specifically, the permutation-based feature importance and the cooperation network among important features in the random forest algorithm were applied to evaluate the relationship of each biomarker to extracellular volume fraction (ECV) in 25 Fontan patients. Among different types of matrix metalloproteinase (MMP) and tissue inhibitor of metalloproteinase (TIMP), MMP-10 and TIMP-1 were the most strongly associated with cardiac fibrosis measured by ECV, suggesting these molecules as promising targets for the prevention and treatment of myocardial fibrosis in Fontan patients. Machine learning may facilitate a more efficient study of biomarkers by identifying the most relevant biomarkers for potential clinical use, thereby enabling biomarker assessment in the context of rare diseases and small sample sizes [117]. Such tools hold promise for guiding the timing of advanced interventions, optimizing surveillance, and informing about the extent of extra-cardiac complications.

### 4.2. The Emerging “-omics” Technologies

In addition to conventional biomarkers, emerging “-omics” technologies—including proteomics, metabolomics, lipidomics, and miRNome—could offer exciting new avenues for understanding Fontan pathophysiology (Figure 3).

These platforms enable the identification of biomolecular patterns that reflect metabolic, inflammatory, and vascular dysfunctions long before overt clinical manifestations occur. For instance, in an initial clinical metabolomics investigation examining serum amino acid profiles in Fontan patients, adult individuals with Fontan circulation demonstrated a dysregulated amino acid metabolome compared to healthy controls [64]. In particular, decreased serum concentrations of taurine, asparagine, and threonine, alongside increased levels of glutamic acid and hydroxyproline in Fontan patients, indicate alterations in energy metabolism and enhanced protein turnover—metabolic features also observed in biventricular patients with congestive HF—in the myocytes. Furthermore, elevated serum concentrations of ADMA, methionine sulfoxide, glutamic acid, and trans-4-hydroxyproline, along with an increased methionine sulfoxide/methionine ratio (Met-SO/Met), suggested a pronounced state of systemic oxidative stress and impaired endothelial function, consistent with subtle ventricular dysfunction. The reduced serum level of histidine—an amino acid with known antioxidant and anti-inflammatory properties—further supports the presence of oxidative stress in Fontan patients [64]. Additionally, the observed reduction in HDL-C levels led the authors to propose a direct interaction between lipoprotein metabolism and oxidative stress markers such as ADMA [118].

To further investigate the lipid and lipoprotein imbalance observed in Fontan circulation, the same cohort of patients was analyzed for small organic molecules within extended phospholipid and acylcarnitine metabolic pathways [87]. The key finding was a significant difference in the concentrations of phosphatidylcholine (PC), sphingomyelin (SM), and acylcarnitine between Fontan patients and controls: Fontan patients exhibited lower total PC and SM levels, alongside elevated total acylcarnitine concentrations. These marked metabolic alterations may reflect disrupted cellular signaling and energy metabolism, chronic low-grade inflammation, and functional or structural abnormalities of the lymphatic or vascular systems, as similarly reported in biventricular heart failure. In a more recent study, the same research group employed an affinity-based proteomics approach targeting cell surface markers, cytokines, and chemokines in serum samples from 20 adult Fontan patients with a well-functioning systemic left ventricle and 20 age- and sex-matched healthy controls, aiming to uncover cellular-level processes specific to Fontan circulation [119]. Analysis of 349 serum proteins revealed four significantly altered protein levels associated with chronic inflammation: Fontan patients exhibited elevated concentrations of syndecan-1 and glycophorin-A, alongside reduced levels of leukemia inhibitory factor (LIF) and nerve growth factor-β (NGF-β), compared to controls. Collectively, these findings indicate that Fontan physiology is characterized by distinct physiological and metabolic instabilities, including persistent low-grade inflammation, oxidative stress dysregulation, and potential damage to cellular structures and translational mechanisms. Notably, a combined panel of proteomics-derived biomarkers and conventional biomarkers (uric acid, GGT, and cholesterol) yielded the highest classification accuracy in distinguishing Fontan patients from controls and may offer valuable insight into the (patho)physiology of this particular circulation. Interestingly, O’Connell et al. (2021) performed a pilot study in control subjects, Fontan patients, and Fontan patients with HF by using a multi-platform metabolomics approach composed of mass spectrometry (MS) and nuclear magnetic resonance (NMR) (which yielded 495 and 26 metabolite measurements, respectively) [120]. The authors found a clear metabolomic signature associated with HF in children and young adults with a single functional ventricle [120].

Novel insights might also emerge from the analysis of the miRNome—the complete profile of microRNA (miRNA) profiling—in Fontan patients. miRNAs are small non-coding RNAs that regulate gene expression post-transcriptionally and play key roles in various biological processes, including inflammation, fibrosis, metabolism, and cardiac remodeling [121]. Investigating miRNA expression patterns in this patient population could reveal novel molecular mechanisms underlying Fontan-associated pathophysiology and potentially identify new biomarkers or therapeutic targets. Recently, in univentricular heart patients with and without Fontan palliation, miR-29b-3p and miR-29c-3p seemed to be markers of advanced liver fibrosis/cirrhosis, suggesting their use in the risk assessment of these patients [122].

Moosmann et al. (2021) reported that PLE in Fontan patients was associated with pronounced immunological disturbances, including severe lymphopenia, T cell deficiency, marked alterations in T cell differentiation, and an increased frequency of regulatory T cells [123]. This immunological profile reflected a state of chronic inflammation and diminished immune protection against pathogens and autoimmunity [123]. These cellular alterations appeared to be driven by dysregulation of multiple miRNA-controlled immunological pathways, highlighting the potential role of miRNAs in modulating immune dysfunction in this population [123]. More recently, Kawamura et al. (2025) found elevated circulating miR-25-3p in pediatric patients with pulmonary arteriovenous malformations after Glenn surgery (G-PAVMs) [124]. Notably, in vitro experiments in human lung microvascular endothelial cells (HMVEC-L) showed that miR-25-3p promoted HMVEC-L angiogenesis, proliferation, and migration under hypoxic conditions through a signaling pathway involving the activation of the Akt/mTOR/HIF-1α axis, leading to angiogenesis under hypoxic conditions. Altogether, these results suggested that miR-25-3p may contribute to G-PAVM development [124].

These findings underscore the potential of “omics”- based approaches not only to identify novel early biomarkers, but also to uncover previously unrecognized pathophysiological mechanisms underlying the diverse complications seen in Fontan circulation. This mechanistic insight could pave the way for the development of targeted therapies, ultimately improving outcomes for this complex and vulnerable patient population. Continued investment in large-scale, collaborative, multi “-omics” research is essential to move the field from descriptive studies toward precision medicine in the management of Fontan physiology.

## 5. Conclusions

The growing interest in identifying biomarkers for the progression of Fontan physiology reflects an increasing awareness within the cardiology community of the challenges posed by this unique and complex population. The investigations conducted thus far represent only a fraction of the potential biomarkers that may prove useful for risk stratification, early detection of complications, and individualized management. However, while these initial findings are promising, the field must now move beyond exploratory analyses.

There is a pressing need for prospective, longitudinal, and multicenter studies to rigorously validate candidate biomarkers and clarify their role in clinical decision-making. As the Fontan population ages and long-term complications become more prevalent, it is essential that research strategies evolve accordingly. Importantly, given the multisystem nature of Fontan-associated pathophysiology—affecting not only the heart but also other organs—future studies should be multidisciplinary in design. Such research should not be confined solely to the domain of cardiology but should actively involve hepatologists, nephrologists, hematologists, and other relevant specialists. A collaborative, integrative approach will be crucial to fully understanding the complex interactions between organ systems in Fontan patients and to developing effective, holistic strategies for surveillance and treatment. Only through this interdisciplinary effort can we hope to improve long-term outcomes and quality of life for this growing patient population.
